# Serum asprosin levels as a potential prognostic biomarker in COVID-19: insights for future respiratory viral pandemics

**DOI:** 10.1186/s12879-025-12227-0

**Published:** 2025-11-24

**Authors:** Esra Erdoğan, Azize Yetişgen

**Affiliations:** 1 Department of Basic Sciences of Pharmacy, Faculty of Pharmacy, Harran University, Yenişehir Campus, Şanlıurfa, Türkiye; 2 Infectious Diseases Clinic, Malatya Training and Research Hospital, Malatya, Türkiye

**Keywords:** Asprosin, COVID-19, Disease severity, Inflammation, SARS-CoV-2

## Abstract

**Background:**

Although the initial surge of the COVID-19 pandemic has passed, identifying prognostic biomarkers remains critical for managing severe cases and preparing for future respiratory viral outbreaks. Asprosin, a metabolic hormone involved in energy homeostasis and inflammation, has been linked to cardiometabolic disorders, but its relationship with COVID-19 severity has not been fully elucidated. This prospective study aimed to investigate the association between serum asprosin levels, disease severity, and clinical outcomes in COVID-19 patients.

**Methods:**

A total of 121 participants were enrolled, including 95 hospitalized patients with RT-PCR–confirmed COVID-19 and 26 PCR-negative controls. Patients were classified into mild, moderate, or severe pneumonia groups based on chest CT findings. Demographic, clinical, and laboratory parameters—including serum asprosin—were analyzed for differences between groups and correlations with disease severity.

**Results:**

Serum asprosin levels were significantly higher in COVID-19 patients compared to controls (*p* < 0.001) and served as an independent predictor of COVID-19 positivity (OR = 2.13, *p* = 0.017). ROC analysis demonstrated strong diagnostic performance (AUC = 0.849, *p* < 0.001), with a cut-off of 4.71 ng/mL yielding 98.9% sensitivity and 69.2% specificity. COVID-19 patients also showed higher ferritin, CRP, and urea, and lower platelet and albumin levels (*p* < 0.001). Mortality was strongly associated with pneumonia severity (*p* < 0.001); low oxygen saturation and hypertension emerged as independent predictors of severity. Asprosin correlated positively with fibrinogen and negatively with comorbidity count. Patients with diabetes mellitus had significantly lower asprosin levels (*p* = 0.042), while no significant differences were observed according to mortality status.

**Conclusions:**

Elevated serum asprosin in COVID-19 patients suggests involvement in metabolic and inflammatory responses. Although unrelated to disease severity, it may aid diagnosis and reflect systemic stress. Larger multicenter studies are needed to confirm its biomarker and therapeutic potential.

**Clinical trial number:**

Not applicable.

**Supplementary Information:**

The online version contains supplementary material available at 10.1186/s12879-025-12227-0.

## Introduction

COVID-19 has emerged as a worldwide health crisis, with over 777 million confirmed cases and more than 7 million fatalities globally [1]. SARS-CoV-2 infection presents with variable severity, from mild respiratory symptoms to critical illness requiring intensive care and mechanical ventilation. Viral replication in respiratory epithelial cells triggers excessive immune activation or “cytokine storm,” leading to acute respiratory distress syndrome (ARDS)—a major cause of mortality—and, in severe cases, multi-organ failure. Advanced age, persistent cough, high fever, chest CT abnormalities, elevated D-dimer levels, obesity, hypertension, and diabetes mellitus (DM) have been identified as predictors of severe disease, hospitalization, and intensive care requirement [2–6].

DM is one of the most prevalent and clinically significant comorbidities in COVID-19, affecting approximately 20–50% of patients. It increases the risk of intensive care admission, complications, and mortality. SARS-CoV-2 infection may worsen pre-existing diabetes and contribute to new-onset diabetes through altered angiotensin-converting enzyme 2 (ACE2) expression. Although optimal glycemic control reduces mortality, pandemic-related lockdowns disrupted diabetes management, leading to poorer outcomes in this vulnerable group [7].

Asprosin, first identified by Romere et al. in 2016, is a peptide hormone encoded by exons 65 and 66 of the fibrillin 1 (FBN1) gene [8]. Secreted by white adipose tissue during fasting, it crosses the blood–brain barrier and stimulates hepatic glucose release via the G protein–cAMP–PKA pathway. Beyond glucose regulation, asprosin modulates energy metabolism, appetite, insulin secretion, inflammatory responses, apoptotic cell death, and reproductive function, with altered signaling implicated in disorders such as DM, obesity, polycystic ovary syndrome, malignancies, and cardiomyopathies [9–12]. As a key adipokine, it is secreted by white adipose tissue through autocrine, paracrine, and endocrine mechanisms [13]. The 30 kDa, 140–amino acid protein is present in multiple tissues and fluids—including adipose tissue, serum, pancreatic β-cells, and ovaries—and exerts its effects via the olfactory receptor OLFR734, which regulates hepatic glucose output and glucose homeostasis [14, 15].

Beyond metabolic regulation, asprosin has been implicated in cardiovascular and respiratory disorders. In our previous rat myocardial infarction (MI) model, elevated serum cytokine levels and brain asprosin expression suggested a role in inflammatory and neuroinflammatory responses. Asprosin elevation correlated with oxidative stress and cytokine imbalance, while antioxidant treatment with nerolidol normalized asprosin and improved inflammation [16]. Similarly, in obstructive sleep apnea syndrome (OSAS), higher serum asprosin levels correlate with disease severity, body mass index, insulin resistance, triglycerides, and the apnea–hypopnea index, and inversely with HDL cholesterol [17]. Collectively, these findings indicate that asprosin may act as a mediator linking metabolic, cardiovascular, and respiratory comorbidities, potentially contributing to systemic inflammatory conditions such as COVID-19.

Given its involvement in both inflammatory pathways and energy metabolism, asprosin may serve as a mediator at the intersection of metabolic and immune dysregulation [18]. COVID-19, characterized by an exaggerated inflammatory response and impaired glucose homeostasis [19], provides a clinical context in which asprosin could influence disease progression. Altered asprosin signaling, previously linked to DM, obesity, and other metabolic disorders, may exacerbate COVID-19 severity through effects on systemic inflammation, insulin sensitivity, and hepatic glucose production. Since COVID-19 disproportionately affects patients with these comorbidities—conditions in which asprosin is already dysregulated—its modulation of glucose metabolism, appetite, and inflammatory signaling [20] may represent a key link between metabolic dysfunction and the hyperinflammatory state observed in COVID-19.

In this context, the present prospective study aimed to investigate circulating asprosin levels in COVID-19 patients and their association with disease severity. To address this, measurements were obtained both at admission and discharge, enabling evaluation of dynamic changes in laboratory and biochemical parameters, including asprosin, during the disease course. This design provides insight into the temporal response of asprosin to infection and treatment, with potential implications for prognosis, therapeutic monitoring, and biomarker discovery.

## Materials and methods

### Study population

This study was conducted at Turgut Özal Training and Research Hospital between April 2022 and December 2022. The study population consisted of 95 hospitalized patients (in wards or intensive care units) with reverse transcriptase–polymerase chain reaction (RT-PCR)–confirmed SARS-CoV-2 infection and a control group of 26 individuals who tested PCR-negative for SARS-CoV-2 and had C-reactive protein (CRP) levels below 1 mg/L, indicating the absence of systemic inflammation. Controls were recruited from the same hospital population during the same study period, and age, sex distribution, and major comorbidities were recorded to ensure comparability with the patient group. Detailed characteristics of both patient and control groups are presented in Table 1. All participants were provided with detailed information about the study, and written informed consent was obtained from those who agreed to participate. The study protocol was approved by the Clinical Research Ethics Committee of Turgut Özal University (Decision No: 2022/45).

### Sample size and power analysis

To determine the adequacy of the sample size, a priori power analysis was performed using the G*Power 3.1.9.2 software, based on the study “Asprosin and Oxidative Stress Level in COVID-19 Patients.” [21]. The analysis indicated that, with a 95% confidence interval and 95% power, a minimum of 30 patients and 10 controls (a total of at least 40 participants) would be sufficient. Considering that the patient group in this study was further stratified into three subgroups, a case-to-control ratio of 3 was applied during the calculation.

### Inclusion and exclusion criteria

Participants were included if they were aged 18 years or older, had a confirmed SARS-CoV-2 infection by RT-PCR (for patients) or a negative PCR with CRP < 1 mg/L (for controls), and were hospitalized with mild, moderate, or severe COVID-19 according to WHO guidelines. Diabetic patients under routine follow-up with internal medicine consultations were also included, although specific data regarding HbA1c levels or diabetes duration were not systematically recorded. All participants were required to have the ability and willingness to provide informed consent.

Exclusion criteria encompassed individuals younger than 18 years, those managed as outpatients for COVID-19, or patients diagnosed with COVID-19 during hospitalization for unrelated conditions. Patients with active infections other than COVID-19 or significant comorbidities that could interfere with study participation—such as malignancy, advanced chronic kidney disease, or advanced liver disease—were also excluded.

### COVID-19 severity classification and patient distribution

The severity of COVID-19 cases was categorized as mild, moderate, or severe in accordance with the World Health Organization (WHO) guidelines [22]. Patients with mild illness were symptomatic but showed no clinical signs of pneumonia and maintained adequate oxygenation (SpO₂ >90% on room air). Moderate illness was defined by the presence of at least one clinical indicator of pneumonia (such as fever, cough, shortness of breath, or rapid breathing) while still maintaining SpO₂ levels above 90% without supplemental oxygen. Severe illness was identified in patients with pneumonia accompanied by either a respiratory rate exceeding 30 breaths per minute or oxygen saturation below 90% on room air.

A total of 95 patients were included in the study. Among them, 28 had mild, 31 moderate, and 36 severe pneumonia. Most patients (*n* = 83) were followed in the ward: 28 mild, 29 moderate, and 26 severe cases, with two of the severe cases managed in the ward due to intensive care unit (ICU) unavailability, both of whom unfortunately died. Additionally, 12 patients were admitted directly to the ICU because of low oxygen saturation and impaired general condition, including 2 with moderate and 10 with severe pneumonia.

### Biochemical and clinical analyses

All biochemical parameters (AST, ALT, CRP, albumin, Hb, ferritin, fibrinogen, PCT, pro-BNP, glucose, etc.) were measured in serum samples in the Biochemistry and Microbiology Laboratories of our hospital. Inflammatory markers specifically assessed in this study included WBC, CRP, PCT, ferritin, fibrinogen, and D-dimer, which are reflective of the systemic inflammatory response in COVID-19 patients. Ferritin was measured by chemiluminescent microparticle immunoassay (Abbott Architect C16000), fibrinogen by the Clauss method (Roche Cobas 8000 E801), CRP by immunoturbidimetric assay (Abbott Architect C16000), D-dimer by immunoturbidimetric assay (Roche Cobas 8000 E801), and pro-BNP by electrochemiluminescence immunoassay (Roche Cobas 8000 E801). For each patient, laboratory and clinical parameters were assessed twice: at hospital admission and prior to discharge.

### Measurement of serum asprosin levels

All blood samples were stored at − 80 °C until analysis. Serum asprosin concentrations were measured during hospitalization using a commercially available ELISA kit (AndyGene, Catalog No: AD12651Hu), following the manufacturer’s instructions. The assay had a detection range of 1–50 ng/L, with a sensitivity of 0.1 ng/L. Optical density was measured at 450 nm using a microplate reader. All samples were analyzed in duplicate, and mean values were used for statistical analysis. The intra-assay and inter-assay coefficients of variation were < 8% and < 10%, respectively, indicating good reproducibility.

### Statistical analysis

Data analysis was performed using SPSS version 22 (Statistical Package for the Social Sciences; IBM Corp., Armonk, NY, USA). Continuous variables were expressed as mean ± standard deviation (SD) or median with interquartile range (IQR, 25th–75th percentiles), depending on data distribution. Categorical variables were presented as frequencies and percentages.

The Kolmogorov–Smirnov test was applied to assess the normality of continuous variables. For comparisons between two independent groups, Student’s t-test was used for normally distributed data, whereas the Mann–Whitney U test was applied for non-normally distributed data. For comparisons involving more than two groups, one-way analysis of variance (ANOVA) was used for normally distributed variables, and the Kruskal–Wallis test was employed for non-normally distributed variables. Paired comparisons before and after hospitalization were conducted using the Wilcoxon signed-rank test. Categorical variables were compared using the chi-square test.

Correlations between continuous variables were assessed using Spearman’s correlation analysis. To identify parameters predicting COVID-19 positivity and severe disease, multivariate logistic regression analysis was performed. Variables that showed statistical significance in univariate comparisons were included in the model, and the Enter method was applied. In addition, multivariate logistic regression models were specifically conducted to evaluate whether asprosin was an independent predictor of COVID-19 severity (mild/moderate vs. severe) and mortality (survivors vs. non-survivors), while adjusting for potential confounders including age, sex, DM, hypertension, and other comorbidities. Receiver operating characteristic (ROC) curve analysis was performed to assess the diagnostic value of asprosin. A p-value less than 0.05 was considered statistically significant.

## Results

### Patient characteristics and clinical symptoms

A total of 121 participants were included: 95 RT-PCR–confirmed COVID-19 patients and 26 controls. The groups were comparable in terms of age and sex distribution (*p* > 0.05). Compared with controls, COVID-19 patients had markedly higher serum asprosin, ferritin, CRP, and urea levels, while platelet counts and albumin were significantly lower (all *p* < 0.001). Minor elevations in AST and ALT were also observed in the patient group. Clinically, 14.7% of patients died, 26.3% were discharged with oxygen support, and 58.9% were discharged without complications (Table 1).

At hospital admission, the dominant symptoms among COVID-19 patients were fatigue, anorexia, and myalgia. Other frequently reported symptoms included shortness of breath (63 patients, 66.32%), cough (61 patients, 64.21%), fever (23 patients, 24.21%), and loss or reduction of smell (32 patients, 33.68%). Eight patients (8.42%) presented with general condition deterioration or syncope.

**Table 1:** Baseline demographic, clinical, and laboratory features of COVID-19 patients and controls

		COVID-19 patients (*n* = 95)		Control (*n* = 26)		*p*
		*n*	%	*n*	%	
Sex	Female	33	34.7	13	50.0	0.155^*^
	Male	62	65.3	13	50.0	
Age, Mean ± SD		66.6 ± 12.5		62.2 ± 17.3		0.229^**^
Number of comorbidities	0	33	34.7	12	46.2	0.491^*^
	1	46	48.4	9	34.6	
	2	14	14.7	5	19.2	
	3	2	2.1	0	0	
Diabetes mellitus	Yes	31	32.6	12	46.2	0.202^*^
	No	64	67.4	14	53.8	
Hypertension	Yes	31	32.6	5	19.2	0.185^*^
	No	64	67.4	21	80.8	
Heart disease	Yes	12	12.6	1	3.8	0.295^*^
	No	83	87.4	25	96.2	
COPD	Yes	5	5.3	1	3.8	0.768^*^
	No	90	94.7	25	96.2	
Asprosin (ng/mL)		7.1 (6.1–9.1)		2.2 (1.6–5.6)		**< 0.001** ^***^
WBC (mcL)		7.2 (5.0-9.7)		7.7 (6.9–8.4)		0.159^***^
Hb (mg/dL)		13.5 (12.3–14.4)		13.5 (12.0–15.0)		0.389^***^
PLT (mcL)		183.0 (147.0-248.0)		288.0 (213.0-321.0)		**< 0.001** ^***^
CRP (mg/dL)		9.5 (4.2–13.2)		0.1 (0-0.5)		**< 0.001** ^***^
Ferritin (ng/mL)		422.0 (227.0-727.0)		28.0 (12.0-81.2)		**< 0.001** ^***^
Urea (mg/dL)		42.0 (31.0–61.0)		31.8 (25.0–39.0)		**0.001** ^***^
Creatinine (mg/dL)		0.9 (0.8–1.3)		0.8 (0.7-1.0)		0.200^***^
AST (U/L)		36.0 (27.0–54.0)		19.0 (16.0–24.0)		**< 0.001** ^***^
ALT (U/L)		23.0 (17.0–40.0)		20.0 (14.0–23.0)		**0.012** ^***^
Albumin (g/dL)		3.1 (2.8–3.4)		4.1 (3.9–4.4)		**< 0.001** ^***^
Exitus	Exitus	14	14.7	-		**-**
	Discharge	56	58.9			
	Discharged with an O_2_ device	25	26.3			

*Data are presented as n (%), mean ± SD, or median (IQR), as appropriate

*Chi-square analysis, **Student t test, **Mann Whitney U test were applied

Abbreviations: ALT: alanine aminotransferase; AST: aspartate transaminase; COPD: chronic obstructive pulmonary disease; CRP: C-reactive protein; Hb: hemoglobin; PLT: platelet count; WBC: white blood cell count

**Fig. 1:**
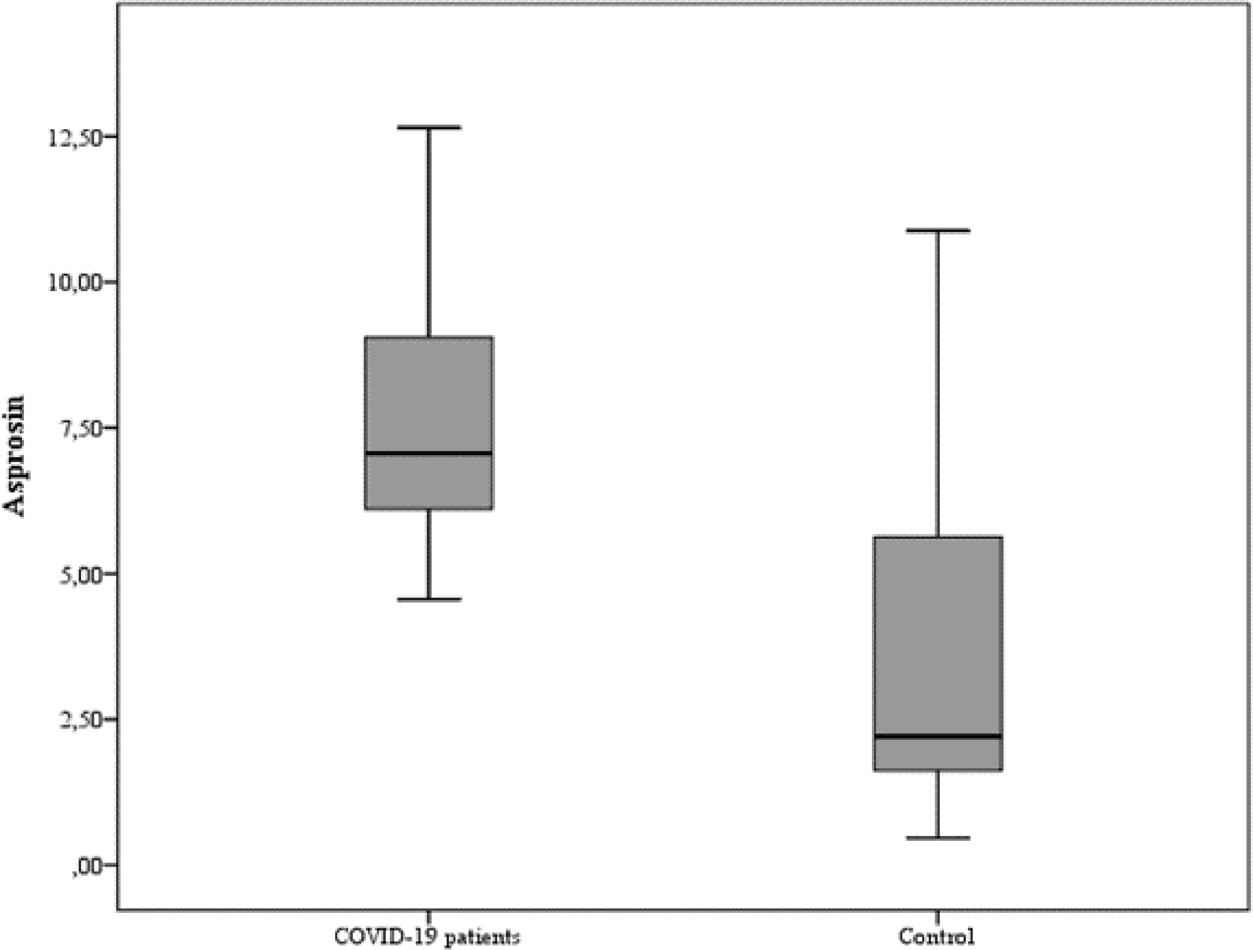
Comparison of serum asprosin levels between COVID-19 patients and controls

Serum asprosin levels were significantly higher in COVID-19 patients compared with the control group (median [IQR]: 7.1 [6.1–9.1] vs. 2.2 [1.6–5.6], *p* < 0.001), as illustrated in Fig. 1.

### Independent predictors of COVID-19 positivity

Logistic regression analysis revealed that elevated asprosin levels significantly increased the likelihood of COVID-19 positivity (OR = 2.130, 95% CI: 1.142–3.971, *p* = 0.017). Conversely, lower platelet counts and reduced albumin levels were also significant predictors (*p* < 0.05). The model demonstrated excellent explanatory power (Nagelkerke R² = 0.874) and correctly classified 95.0% of cases, underscoring the potential role of asprosin as a novel biomarker in COVID-19 (Table 2).

**Table 2:** Multivariate logistic regression analysis for predictors of COVID-19 positivity

	B	S.E.	*p*	OR	95% C.I.for EXP (B)	
					Lower	Upper
Asprosin	0.756	0.318	**0.017**	2.130	1.142	3.971
PLT	-0.019	0.009	**0.030**	0.981	0.964	0.998
Albumin	-7.302	2.137	**0.001**	0.002	0.001	0.044

Abbreviations: B: regression coefficient; EXP(B): exponentiated B (odds ratio); OR: odds ratio; PLT: platelet count; SE: standard error

### ROC analysis for asprosin as a predictor of COVID-19

**Fig. 2:**
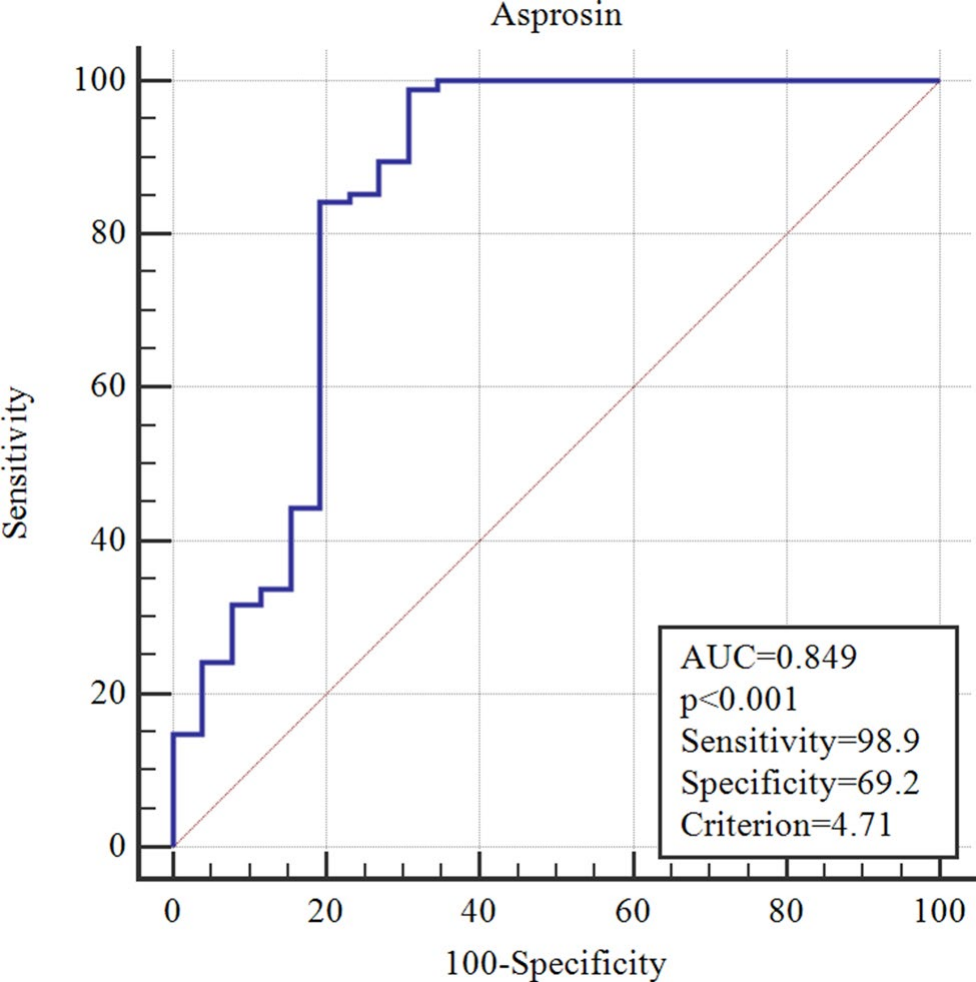
ROC curve of asprosin levels for predicting the presence of COVID-19

The ROC curve revealed that asprosin is a strong predictor of COVID-19, with an AUC of 0.849 (*p* < 0.001). Using a cut-off value of 4.71 ng/mL, asprosin achieved very high sensitivity (98.9%) with acceptable specificity (69.2%), highlighting its potential as a clinically valuable biomarker (Fig. 2).

### Clinical outcomes according to pneumonia severity

Of the 95 COVID-19 patients, 29.5% had mild, 32.6% moderate, and 37.9% severe pneumonia on chest CT. Mortality increased significantly with disease severity (*p* < 0.001): no deaths occurred in the mild group, whereas mortality reached 12.9% in moderate cases and 27.8% in severe cases. Moreover, the need for supplemental oxygen at discharge was substantially higher in patients with severe pneumonia (41.7%) compared to moderate cases (32.3%), while all mild cases recovered without complications (Table 3).

**Table 3:** Comparison of demographic data, presence of DM and exitus status in the patient group according to CT findings

		Mild (*n* = 28)		Moderate (*n* = 31)		Severe (*n* = 36)		p*
		Number	%	Number	%	Number	%	
Sex^a^	Female	9	27.3	14	42.4	10	30.3	0.311
	Male	19	30.6	17	27.4	26	41.9	
Age, Mean ± SD		62.3 ± 13.4		67.4 ± 13.7		69.4 ± 9.8		0.070^**^
DM^a^	Yes	9	29.0	11	35.5	11	35.5	0.910
	No	19	29.7	20	31.3	25	39.1	
Exitus^b^	Exitus	0	0	4	12.9	10	27.8	**< 0.001**
	Discharge	28	100	17	54.8	11	30.6	
	Discharged with an O_2_ device	0	0	10	32.3	15	41.7	

^*^Chi-square analysis, **One Way ANOVA test was applied. ^a^Row percentage, ^b^Column percentage are given

### Comparison of laboratory parameter changes according to pneumonia severity

Laboratory changes varied significantly with pneumonia severity. Patients with severe pneumonia had smaller reductions in hemoglobin and platelet counts compared to those with mild or moderate disease (*p* ≤ 0.001). ALT changes were most pronounced in mild cases (*p* = 0.015). Oxygen saturation declined progressively with increasing severity, with the greatest decrease observed in severe pneumonia (*p* < 0.001). Detailed pairwise comparisons are provided in Table 4.

**Table 4:** Comparison of changes in laboratory parameters according to pneumonia severity based on CT findings

	Mild Median (IQR)	Moderate Median (IQR)	Severe Median (IQR)	p*
Asprosin (ng/mL)	-1.0 (-1.1-0.6)	-0.3 (-21-0.3)	-1.0 (-2.4-0.2)	0.525
WBC (mcL)	3.9 (0.8–5.6)	4.9 (2.5–6.1)	3.0 (0.4–6.8)	0.299
Hb (mg/dL)	-0.9 (-1.3–0.3)^a^	− 0.4 (-1.2-0.3)^a^	-1.5 (-2.4–0.6)^b^	**0.001**
PLT (mcL)	135.0 (93.5-194.5)^a^	110.0 (40.0-200.0)^a^	-0.5 (-31.0-89.0)^b^	**< 0.001**
CRP (mg/dL)	-8.7 (-10.5–3.1)	-6.2 (-11.0–37)	-7.0 (-13.4–4.5)	0.630
PCT (ng/mL)	-0.1 (-0.2-0.0)	0.0 (-0.2-0.0)	-0.1 (-0.3-0.0)	0.344
Ferritin (ng/mL)	-4.5 (-41.5-181.5)	-6.0 (-78.0-164.0)	-14.0 (-217.0-344.5)	0.850
Fibrinogen (mg/dL)	-122.0 (-161.0–51.0)	-77.0 (-168.0–4.0)	-126.0 (-303.5–37.5)	0.245
D-dimer (µg/mL)	0.0 (-0.3-0.3)	-0.2 (-0.6-0.0)	0.0 (-0.6-1.2)	0.605
Pro-BNP (pg/mL)	-16.0 (-208.0-58.0)	-148.0 (-431.0-30.0)	-117.0 (-975.0-977.0)	0.822
PT (seconds)	-0.8 (-1.0-0.1)	0.1 (-1.0-0.5)	0.0 (-1.8-1.5)	0.500
aPTT (seconds)	-2.4 (-3.9–0.1)	-1.3 (-5.3-3.1)	-2.2 (-5.4–0.3)	0.554
INR (ratio)	-0.1 (-0.1-0.0)	0.0 (-0.1-0.1)	0.0 (-0.2-0.1)	0.480
Glucose (mg/dL)	17.0 (-7.0-57.0)	3.0 (-40.0-55.0)	-1.0 (-35.0-63.0)	0.342
Urea (mg/dL)	12.0 (-1.0-20.5)	7.0 (-2.0-16.0)	4.0 (-13.0-17.0)	0.353
Creatine (mg/dL)	-0.2 (-0.4-0.0)	-0.1 (-0.3-0.0)	-0.2 (-0.3-0.0)	0.770
AST (U/L)	-7.5 (-16.5-2.5)	-11.0 (-29.0–1.0)	-13.5 (-29.5–6.0)	0.096
ALT (U/L)	2.0 (16.0–56.0)^a^	9.0 (-2.0-21.0)^b^	6.5 (-2.5-29.5)^b^	**0.015**
Albumin (g/dL)	-0.4 (-0.8-0.1)	-0.4 (-0.7–0.1)	-0.4 (-0.8-0.0)	0.752
O_2_ Saturation (%)	6.0 (5.0-6.5)^a^	7.0 (6.0–10.0)^b^	10.0 (6.0-13.5)^c^	**< 0.001**

^*^Kruskal–Wallis test was applied. ^a, b, c^: Different superscript letters indicate significant pairwise differences

Abbreviations: ALT: alanine aminotransferase; AST: aspartate transaminase; aPTT: activated partial thromboplastin time; CRP: C-reactive protein; Hb: hemoglobin (mg/dL); O₂ Saturation: oxygen saturation; PLT: platelet count; Pro-BNP: pro-brain natriuretic peptide; PCT: procalcitonin; PT: prothrombin time; WBC: white blood cell count; INR: international normalized ratio

### Independent predictors of severe pneumonia

In the logistic regression analysis, only oxygen saturation (OR = 0.75; 95% CI: 0.63–0.90; *p* = 0.001) and the presence of hypertension (OR = 4.04; 95% CI: 1.24–13.12; *p* = 0.020) were found to be independently significant. Other variables (exitus, Hb, PLT, ALT, heart disease) did not show a statistically significant contribution (*p* > 0.05) (Table 5).

**Table 5:** Multivariate logistic regression analysis results predicting severe pneumonia

	B	S.E.	Sig.	Exp (B)	95% C.I.for EXP (B)	
					Lower	Upper
Exitus	0.436	0.832	0.601	1.546	0.303	7.903
Hb	0.155	0.187	0.408	1.167	0.809	1.685
PLT	0.000	0.004	0.987	1.000	0.992	1.008
ALT	0.012	0.013	0.374	1.012	0.986	1.039
O₂ Saturation	-0.286	0.090	**0.001**	0.751	0.630	0.896
Hypertension	1.396	0.601	**0.020**	4.039	1.243	13.122
Heart Disease	1.748	0.958	0.068	5.745	0.879	37.543

Abbreviations: ALT: alanine aminotransferase; B: regression coefficient; Exp(B): exponentiated B (odds ratio); Hb: hemoglobin; PLT: platelet count; SE: standard error

### Changes in blood parameters during hospitalization in COVID-19 patients

Before discharge, the levels of asprosin, hemoglobin, CRP, PCT, fibrinogen, pro-BNP, INR, aPTT, creatinine, CK, AST, and albumin decreased significantly in the patient group. In contrast, the WBC, PLT, urea, ALT, and saturation values increased significantly (Table 6).

**Table 6:** Comparison of changes in blood parameters in the patient group

	During Hospitalization	Before Discharge	*p* ^*^
	Median (IQR)	Median (IQR)	
Asprosin (ng/mL)	7.1 (6.1–9.1)	6.7 (6.2–7.3)	**< 0.001**
WBC (mcL)	7.2 (5.0-9.7)	10.6 (7.9–14.0)	**< 0.001**
Hb (mg/dL)	13.5 (12.3–14.4)	12.3 (11.3–13.8)	**< 0.001**
PLT (mcL)	183.0 (147.0-248.0)	275.0 (206.0-369.0)	**< 0.001**
CRP (mg/dL)	9.5 (4.2–13.2)	0.5 (0.1–2.5)	**< 0.001**
PCT (ng/mL)	0.2 (0.1–0.3)	0.1 (0-0.1)	**< 0.001**
Ferritin (ng/mL)	422.0 (227.0-727.0)	444.0 (240.0-816.0)	0.276
Fibrinogen (mg/dL)	442.0 (357.5–512.0)	326.0 (266.0-384.0)	**< 0.001**
D-dimer (µg/mL)	0.7 (0.4–1.3)	0.6 (0.4–1.4)	0.522
Pro-BNP (pg/mL)	446.0 (173.0-1599.0)	278.0 (99.0-1064.0)	**0.026**
PT (seconds)	11.9 (11.0-12.6)	11.7 (10.7–12.6)	0.087
aPTT (seconds)	26.0 (23.5–28.7)	24.1 (21.3–27.2)	**< 0.001**
INR (ratio)	1.1 (1.0-1.2)	1.0 (1.0-1.1)	**0.016**
Glucose (mg/dL)	132.0 (112.0-170.0)	149.0 (102.0-242.0)	0.116
Urea (mg/dL)	42.0 (31.0–61.0)	52.0 (38.0–70.0)	**< 0.001**
Creatine (mg/dL)	0.9 (0.8–1.3)	0.8 (0.7-1.0)	**< 0.001**
AST (U/L)	36.0 (27.0–54.0)	25.0 (18.0–34.0)	**< 0.001**
ALT (U/L)	23.0 (17.0–40.0)	42.0 (27.0–69.0)	**< 0.001**
Albumin (g/dL)	3.1 (2.8–3.4)	2.7 (2.4-3.0)	**< 0.001**
O_2_ Saturation (%)	86.0 (84.0–89.0)	94.0 (92.0–95.0)	**< 0.001**

^*^ Wilcoxon analysis was applied

Abbreviations: ALT: alanine aminotransferase; aPTT: activated partial thromboplastin time; AST: aspartate transaminase; CRP: C-reactive protein; Hb: hemoglobin; O₂ Saturation: oxygen saturation; PLT: platelet count; Pro-BNP: pro-brain natriuretic peptide; PCT: procalcitonin; PT: prothrombin time; WBC: white blood cell count

## Discussion

The clinical severity of COVID-19 varies widely, ranging from mild symptoms to severe pneumonia, ARDS, and multi-organ involvement. Patient-specific factors, including age, sex, and comorbidities such as DM, hypertension, and obesity, strongly influence outcomes. Laboratory markers reflecting systemic inflammation and tissue/organ injury—such as CRP, ferritin, fibrinogen, and ProBNP—correlate with disease severity and mortality [23]. Identifying biomarkers that integrate both metabolic and inflammatory status could improve early risk stratification and guide patient management.

In this context, our prospective cohort study demonstrated that serum asprosin levels were significantly higher in COVID-19 patients compared to controls. During hospitalization, asprosin correlated positively with fibrinogen and disease severity markers, whereas diabetic patients exhibited lower levels than non-diabetics. These results suggest that asprosin may serve as a potential biomarker reflecting both inflammatory and metabolic stress in COVID-19, providing additional insight into the disease’s complex pathophysiology.

SARS-CoV-2 enters host cells through its spike protein by binding to ACE2 receptors, which are expressed not only in the lungs but also in the small intestine, adipose tissue, kidneys, heart, thyroid, and testis. Adipose tissue, with high ACE2 expression, may serve as a viral reservoir, and postmortem analyses have revealed increased inflammation in fat tissue as well as fat embolisms in the lungs and liver of COVID-19 patients [24]. In this context, asprosin, a glycogenic hormone secreted by white adipose tissue during fasting, plays a key role in energy homeostasis by stimulating hepatic glucose production and modulating appetite via AgRP neurons. Circulating asprosin levels are elevated in diabetes, obesity, and other cardiometabolic disorders and have been associated with proinflammatory effects through cytokine and chemokine induction [25, 26]. These metabolic and inflammatory roles, together with its correlation with body mass index (BMI) in DM, support asprosin as a potential biomarker linking adipose tissue status to COVID-19 outcomes.

Several mechanisms may explain the observed elevation of serum asprosin in COVID-19 patients. First, asprosin, secreted primarily by white adipose tissue during fasting, regulates hepatic glucose release and insulin sensitivity [27]. COVID-19 is characterized by systemic inflammation and metabolic dysregulation, including stress-induced hyperglycemia [28], and elevated asprosin may represent a compensatory response to maintain energy homeostasis. Second, asprosin exerts pro-inflammatory effects through the TLR4/NF-κB pathway, stimulating cytokines such as TNF-α and IL-6 [29, 30]. Given the critical role of cytokine dysregulation in COVID-19 severity, higher asprosin levels may contribute to or amplify systemic inflammation. Third, comorbidities linked to severe COVID-19, such as obesity, diabetes, and metabolic syndrome [31], are also associated with elevated circulating asprosin [32]. Together, these findings suggest that increased serum asprosin in COVID-19 may result from the combined effects of systemic inflammation, altered energy metabolism, and underlying metabolic conditions, linking it to disease severity and prognosis.

Patients with uncontrolled DM are at higher risk for severe outcomes following SARS-CoV-2 infection due to vascular complications and impaired pulmonary function. Even short-term hyperglycemia can induce a pro-inflammatory state, exacerbating SARS-CoV-2 infection and contributing to cytokine storm, lung injury, ICU admission, and mortality [33]. Experimental studies indicate that asprosin can trigger proinflammatory responses in THP-1 macrophages, increasing secretion of TNF-α, IL-1β, IL-6, IL-8, and IL-12 [29, 34], and promotes hyperlipidemia-induced endothelial inflammation via NF-κB signaling [30]. In our cohort, disease severity correlated with glucose levels, but not with asprosin overall, likely reflecting comorbidity heterogeneity. Notably, in diabetic patients, disease severity correlated significantly with both glucose (*r* = 0.472, *p* = 0.007) and asprosin (*r* = 0.562, *p* = 0.001), while glucose and asprosin were not directly correlated (*r* = 0.152, *p* = 0.414), suggesting independent contributions to disease severity.

Among the 95 COVID-19 patients, 28 had mild, 31 moderate, and 36 severe pneumonia. Severe pneumonia on chest CT strongly correlated with mortality and oxygen requirement, with low oxygen saturation and hypertension emerging as independent predictors of disease severity. Longitudinal laboratory analyses showed significant decreases in asprosin, Hb, CRP, PCT, fibrinogen, pro-BNP, INR, aPTT, creatinine, AST, and albumin, while WBC, platelet count, urea, ALT, and oxygen saturation increased, likely reflecting both the resolution of systemic inflammation and the effects of supportive care. Correlation analyses revealed a positive association between serum asprosin and fibrinogen, and a negative association with comorbidity burden. Disease severity correlated positively with age, comorbidity burden, WBC, PCT, fibrinogen, pro-BNP, glucose, and urea, and negatively with albumin and oxygen saturation. Diabetic patients had lower asprosin levels, whereas sex, hypertension, cardiovascular disease, COPD, pneumonia severity, or mortality did not significantly influence asprosin concentrations. Survival analysis indicated that older age, elevated WBC, and urea were associated with mortality, while asprosin levels tended to be higher in non-survivors, without reaching statistical significance.

Serum asprosin levels significantly decreased from hospitalization to pre-discharge measurements, whereas no differences were observed across CT-defined pneumonia severity groups, suggesting that asprosin primarily reflects dynamic changes during acute disease progression rather than static lung involvement. These findings indicate that early measurement of asprosin could help identify high-risk patients, and tracking its levels during hospitalization may provide valuable insights into disease progression and response to treatment. Given its dual role in metabolic regulation and inflammation, asprosin may serve as a prognostic biomarker and potential therapeutic target, although validation in larger, prospective cohorts is warranted.

### Temporal dynamics of asprosin levels in COVID-19

In this prospective study, serum asprosin levels were significantly elevated in COVID-19 patients compared to controls and correlated positively with fibrinogen and disease severity markers, while diabetic patients showed lower levels than non-diabetics. Asprosin independently predicted infection (AUC = 0.849; cut-off 4.71 ng/mL; sensitivity 98.9%, specificity 69.2%), highlighting its potential as a diagnostic biomarker. These results suggest that asprosin reflects the inflammatory and metabolic stress characteristic of COVID-19. Consistent with previous reports, patients also showed increased ferritin, CRP, and urea, alongside reduced platelet counts and albumin, whereas mild elevations in AST and ALT indicated modest hepatic involvement [35, 36].

During hospitalization, several laboratory parameters changed dynamically. Levels of asprosin, CRP, PCT, fibrinogen, pro-BNP, and albumin decreased, while WBC, platelets, urea, ALT, and oxygen saturation increased, reflecting partial resolution of systemic inflammation and metabolic stress. Notably, asprosin declined significantly but did not differ across CT-defined pneumonia severity groups, suggesting that it mirrors systemic stress rather than the extent of lung involvement and may serve as a dynamic biomarker of overall disease progression.

In a study by Karagöz and Aydın involving four groups of COVID-19 patients (each consisting of 22 individuals with varying oxygen saturation levels), serum asprosin levels were reported to increase as oxygen saturation decreased [37]. Consistent with our observations, their study found significantly higher asprosin concentrations in COVID-19 patients compared to controls. In our cohort, asprosin levels also tended to rise with decreasing oxygen saturation in both diabetic and non-diabetic patients, likely reflecting the underlying inflammatory response. While there was a trend toward higher asprosin levels with increasing disease severity, this increase did not reach statistical significance in our cohort, highlighting potential variability due to comorbidities, age, or other patient-specific factors.

In contrast, Seyhanlı et al. [21] reported lower asprosin levels in COVID-19 patients compared to healthy controls. These seemingly contradictory findings may be attributed to differences in patient populations, disease stage at sampling, and assay methods. Seyhanlı’s study included relatively young individuals without systemic or metabolic comorbidities presenting to the emergency department, whereas all patients in our study were hospitalized with severe COVID-19, had a higher mean age, and frequently had multiple comorbidities. By considering these factors, our study clarifies the relationship between asprosin levels and COVID-19 severity in a hospitalized, comorbidity-rich population.

Wen et al. [38] conducted a five-year prospective follow-up of 50 patients with dilated cardiomyopathy and found that higher serum asprosin levels were associated with better cardiovascular outcomes, suggesting a potential protective role under hypoxic conditions. Consistent with this, in our cohort, serum asprosin levels significantly decreased after hospitalization as patients’ oxygen saturation improved. Notably, patients discharged with supplemental oxygen had relatively higher asprosin levels compared to those not requiring oxygen, which may reflect a compensatory response aimed at maintaining oxygen homeostasis under persistent hypoxic stress.

COVID-19 often triggers coagulopathy and thrombotic complications due to its profound impact on the coagulation system. Fibrinogen, a positive acute-phase reactant that increases during systemic inflammation, is strongly associated with disease severity and ICU admission [39–41]. In our cohort, serum asprosin levels positively correlated with fibrinogen, suggesting a potential link between metabolic regulation and inflammatory pathways. Although this correlation does not establish causality, prior studies indicate that asprosin can modulate cytokine production and interact with metabolic processes [42], providing a plausible biological explanation. Further research is needed to elucidate the mechanistic relationship between asprosin, coagulation, and systemic inflammation in COVID-19.

Taken together, these findings suggest that early measurement of asprosin upon hospital admission could help identify high-risk patients, enabling closer monitoring and timely interventions. Additionally, tracking dynamic changes in asprosin levels during hospitalization may provide valuable insights into disease progression and treatment response. Given its dual roles in metabolic regulation and inflammation, asprosin could also represent a promising therapeutic target. However, validation in larger, prospective, and more diverse cohorts is necessary to determine optimal cut-off values, compare its predictive power with established biomarkers, and clarify its clinical utility.

### Clinical implications of asprosin in COVID-19

Our findings suggest that serum asprosin may have meaningful clinical utility in COVID-19 management. Elevated asprosin levels at hospital admission were independently associated with infection and showed strong diagnostic performance (AUC = 0.849). Its positive correlation with fibrinogen and negative correlation with comorbidity burden indicate that asprosin reflects both inflammatory and metabolic stress. Measuring asprosin could support patient triage and facilitate early identification of those at greater risk for severe disease or need for supplemental oxygen. Moreover, asprosin may complement established biomarkers such as CRP, ferritin, and D-dimer in guiding timely interventions and monitoring treatment response.

### Limitations and future perspectives

This study has several limitations. First, BMI data were unavailable; however, none of the participants—including both COVID-19 patients and controls—were obese, which may minimize adiposity-related confounding effects on serum asprosin levels. Second, data on hospitalization duration were incomplete, and as a single-center study, generalizability may be limited. Despite these constraints, this research provides novel insights into the multifaceted role of asprosin in COVID-19, particularly its associations with disease severity, glucose levels, and oxygen saturation. Future multicenter studies with larger cohorts and detailed metabolic profiling are warranted to further elucidate these relationships and validate asprosin’s diagnostic and prognostic potential.

## Conclusion

Although COVID-19 mortality has declined compared to earlier pandemic stages, it remains a significant global health concern due to ongoing viral circulation and emerging variants. In this study, serum asprosin levels were associated with inflammatory markers, oxygen saturation, and several clinical outcomes in hospitalized patients, underscoring its potential involvement in COVID-19 pathophysiology. Collectively, these findings support the utility of asprosin as a prognostic biomarker that may aid in patient management and risk stratification. Future multicenter studies with well-characterized, diverse cohorts and comprehensive metabolic profiling are essential to confirm these findings and explore the potential of asprosin as both a prognostic indicator and a therapeutic target in COVID-19 and other inflammatory respiratory diseases.

## Supplementary Information

Below is the link to the electronic supplementary material.

Supplementary Material 1

## Data Availability

The data supporting the findings of this study are available from the corresponding author on reasonable request.

## Declarations

### Declaration of AI and AI-assisted technologies in the writing process

During the preparation of this work, the authors used the software ChatGPT to enhance readability and language quality. Following the use of this tool, the authors carefully reviewed and edited the content as necessary and take full responsibility for the final content of the publication.

## Abbreviations

ALT: Alanine aminotransferase
aPTT: Activated partial thromboplastin time
AST: Aspartate transaminase
COPD: chronic obstructive pulmonary disease
CRP: C-reactive protein
CT: Computed tomography
DM: Diabetes mellitus
Hb: hemoglobin
HT: Hypertension
INR: International normalized ratio
PCT: Procalcitonin
PLT: Platelet count
Pro-BNP: Pro-brain natriuretic peptide
PT: Prothrombin time
WBC: White blood cell
