# Supplementary material for: Serum asprosin levels as a potential prognostic biomarker in COVID-19: insights for future respiratory viral pandemics
Source: BMC Infect Dis. 2025 Nov 24;25:1809. doi: 10.1186/s12879-025-12227-0 (PMC12751601; doi:10.1186/s12879-025-12227-0)
Supplement: Supplementary file 1 — Supplementary Material 1 [file 12879_2025_12227_MOESM1_ESM.docx]

**Correlation of Asprosin Levels with Clinical and Laboratory Parameters**

In COVID-19 patients, higher prehospitalization asprosin levels correlated positively with fibrinogen and negatively with the number of comorbidities. Disease severity was associated with older age, greater comorbidity burden, and elevated inflammatory and metabolic markers (WBC, PCT, fibrinogen, pro-BNP, glucose, urea), while lower albumin and oxygen saturation were inversely related to severity (Table 7).

**Table 7.** Correlation of Asprosin Levels During Hospitalization with COVID-19 Severity, Age, Comorbidities, and Laboratory Parameters in Patients.

|  | | **Asprosin** | **Severity of COVID-19** |
| --- | --- | --- | --- |
| **Severity of COVID-19** | r | .122 |  |
|  | p | .239 |  |
| **Age** | r | -.011 | **.208** |
|  | p | .914 | **.043** |
| **Number of comorbidities** | r | **-.256** | **.332** |
|  | p | **.012** | **.001** |
| **WBC** | r | .074 | **.358** |
|  | p | .478 | **.000** |
| **Hb** | r | -.097 | .108 |
|  | p | .352 | .296 |
| **PLT** | r | .070 | .151 |
|  | p | .501 | .145 |
| **CRP** | r | .078 | .189 |
|  | p | .454 | .066 |
| **PCT** | r | -.085 | **.267** |
|  | p | .414 | **.009** |
| **Ferritin** | r | .038 | .112 |
|  | p | .718 | .278 |
| **Fibrinogen** | r | **.215** | **.261** |
|  | p | **.040** | **.012** |
| **d dimer** | r | -.004 | .137 |
|  | p | .970 | .186 |
| **Pro-BNP** | r | -.066 | **.333** |
|  | p | .534 | **.001** |
| **PT** | r | -.006 | .098 |
|  | p | .956 | .345 |
| **aPTT** | r | -.064 | .026 |
|  | p | .539 | .803 |
| **INR** | r | -.077 | .039 |
|  | p | .459 | .705 |
| **Glucose** | r | -.012 | **.251** |
|  | p | .908 | **.014** |
| **Urea** | r | .001 | **.257** |
|  | p | .996 | **.012** |
| **Creatine** | r | -.145 | -.077 |
|  | p | .160 | .458 |
| **AST** | r | .084 | .085 |
|  | p | .416 | .411 |
| **ALT** | r | .097 | .143 |
|  | p | .351 | .168 |
| **Albumin** | r | -.081 | **-.306** |
|  | p | .433 | **.003** |
| **O_2_ Saturation** | r | -.092 | **-.706** |
|  | p | .374 | **.000** |

As shown in Figure 3, asprosin levels were positively correlated with both the number of comorbidities and fibrinogen levels.


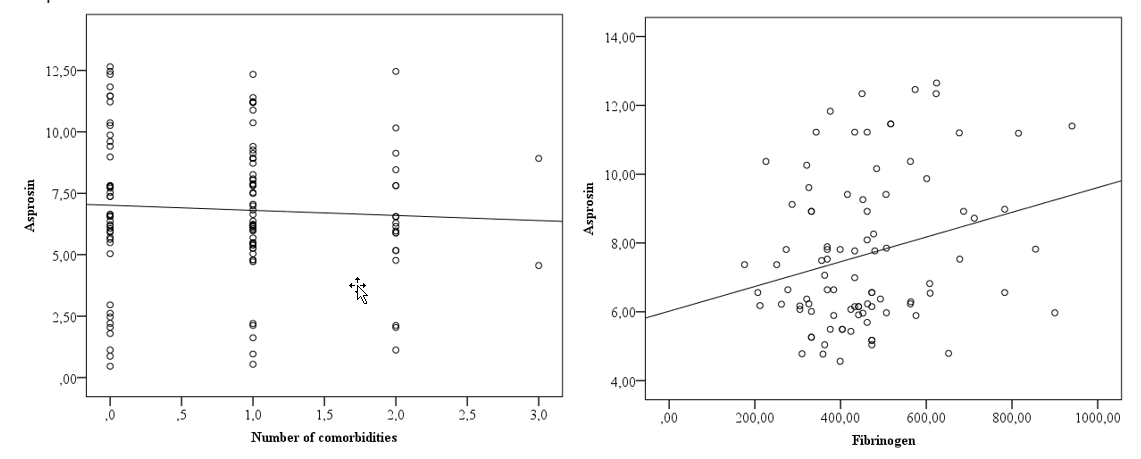


**Figure 3.** Correlation of asprosin with the number of comorbidities and fibrinogen

Asprosin levels measured before discharge showed a significant positive correlation with CRP (r = 0.225; p = 0.028), ferritin (r = 0.264; p = 0.010), and fibrinogen (r = 0.229; p = 0.028), and a significant negative correlation with the number of comorbidities (r = -0.206; p = 0.045).

**Comparison of Serum Asprosin Levels According to Clinical and Demographic Parameters**

Additionally, individuals with diabetes showed significantly lower serum asprosin levels compared to those without diabetes (p=0.042). Table 6 presents the comparison of serum asprosin levels before hospitalization according to different clinical and demographic parameters, highlighting the impact of DM status on asprosin concentrations (Table 8).

**Table 8.** Comparison of serum asprosin levels before hospitalization according to various parameters

|  |  | **Asprosin** | **p** |
| --- | --- | --- | --- |
|  |  | **Median (IQR)** |  |
| **Sex** | Female | 7.5 (6.2-9.3) | 0.326^*^ |
|  | Male | 6.8 (6.0-8.9) |  |
| **DM** | Yes | 6.2 (5.9-7.9) | **0.042^*^** |
|  | No | 7.5 (6.2-9.7) |  |
| **HT** | Yes | 6.8 (5.7-8.9) | 0.168^*^ |
|  | No | 7.4 (6.2-9.5) |  |
| **Heart disease** | Yes | 7.7 (6.0-10.7) | 0.691^*^ |
|  | No | 6.8 (6.1-9.0) |  |
| **COPD** | Yes | 6.2 (6.0-6.3) | 0.191^*^ |
|  | No | 7.4 (6.2-9.3) |  |
| **Exitus** | Exitus | 7.7 (6.0-11.2) | 0.715^**^ |
|  | Discharge | 7.2 (6.1-9.4) |  |
|  | Discharged with an O_2_ device | 6.8 (6.2-7.9) |  |
| **CT** | Mild | 6.6 (5.6-8.4) | 0.495^**^ |
|  | Moderate | 7.4 (6.2-9.1) |  |
|  | Severe | 7.8 (6.1-10.3) |  |

*Mann Whitney U test, **Kruskal Wallis analysis was applied.

**Comparison of Demographic and Laboratory Parameters According to Survival**

As shown in Table 9, non-survivors were significantly older than survivors (p = 0.030). In addition, key laboratory parameters including WBC count (p = 0.005) and serum urea levels (p = 0.004) were significantly higher in non-survivors compared to survivors.

**Table 9.** Comparison of general characteristics according to survival status

|  | | **Exitus yes**  **(n=14)** | **Exitus no**  **(n=81)** | **p** |
| --- | --- | --- | --- | --- |
|  | | n (%) | n (%) |  |
| **Sex** | Female | 3 (%9.1) | 30 (%90.9) | 0.366^*^ |
|  | Male | 11 (%17.7) | 51 (%82.3) |  |
| **Age, Mean ± SD** | | 73.3±13.1 | 65.5±12.1 | **0.030^**^** |
| **Number of comorbidities** | 0 | 3 (%9.1) | 30 (%90.9) | 0.132^*^ |
|  | 1 | 6 (%13.0) | 40 (%87.0) |  |
|  | 2 | 5 (%35.7) | 9 (%64.3) |  |
|  | 3 | 0 (%.0) | 2 (%100.0) |  |
| **Diabetes mellitus** | Yes | 4 (%12.9) | 27 (%87.1) | 0.726^*^ |
|  | No | 10 (%15.6) | 54 (%84.4) |  |
| **Hypertension** | Yes | 7 (%22.6) | 24 (%77.4) | 0.215^*^ |
|  | No | 7 (%10.9) | 57 (%89.1) |  |
| **Heart disease** | Yes | 4 (%33.3) | 8 (%66.7) | 0.074^*^ |
|  | No | 10 (%12.0) | 73 (%88.0) |  |
| **COPD** | Yes | 1 (%20.0) | 4 (%80.0) | 0.558^*^ |
|  | No | 13 (%14.4) | 77 (%85.6) |  |
| **Asprosin (ng/mL)** | | 7.7 (6.0-11.2) | 7.0 (6.2-9.0) | 0.532^***^ |
| **WBC (mcL)** | | 9.3 (7.2-13.2) | 6.8 (4.8-8.5) | **0.005^***^** |
| **Hb (mg/dL)** | | 13.4 (12.3-14.2) | 13.5 (12.4-14.4) | 0.686^***^ |
| **PLT (mcL)** | | 222.5 (180.0-267.0) | 178.0 (142.0-239.0) | 0.105^***^ |
| **CRP (mg/dL)** | | 9.9 (3.4-18.4) | 9.5 (4.9-12.0) | 0.600^***^ |
| **Ferritin (ng/mL)** | | 355.0 (171.0-735.0) | 438.0 (232.0-700.0) | 0.466^***^ |
| **Urea (mg/dL)** | | 59.0 (48.0-78.0) | 39.0 (30.0-58.0) | **0.004^***^** |
| **Creatinine (mg/dL)** | | 1.2 (0.8-1.4) | 0.9 (.8-1.2) | 0.144^***^ |
| **AST (U/L)** | | 38.0 (27.0-53.0) | 36.0 (27.0-54.0) | 0.644^***^ |
| **ALT (U/L)** | | 24.0 (14.0-33.0) | 23.0 (17.0-41.0) | 0.409^***^ |
| **Albumin (g/dL)** | | 3.2 (2.7-3.7) | 3.1 (2.8-3.4) | 0.581^***^ |
| **Glucose (mg/dL)** | | 141.0 (116.0-193.0) | 132.0 (112.0-158.0) | 0.644^***^ |

# *Data are presented as n (%), mean ± SD, or median (IQR), as appropriate.
